# A glimpse into the foraging and movement behaviour of *Nyctalus aviator*; a complementary study by acoustic recording and GPS tracking

**DOI:** 10.1098/rsos.230035

**Published:** 2023-06-28

**Authors:** Yoshifumi Niga, Emyo Fujioka, Olga Heim, Akito Nomi, Dai Fukui, Shizuko Hiryu

**Affiliations:** ^1^ Faculty of Life and Medical Sciences, Doshisha University, 1-3 Tatara miyakodani, Kyotanabe, Kyoto 610-0321, Japan; ^2^ Organization for Research Initiatives and Development, Doshisha University, 1-3 Tatara miyakodani, Kyotanabe, Kyoto 610-0321, Japan; ^3^ The University of Tokyo Hokkaido Forest, Graduate School of Agricultural and Life Sciences, The University of Tokyo, 9-61, Yamabe-Higashimachi, Furano, Hokkaido 079-1563, Japan

**Keywords:** bat, bio-logging, echolocation, microphone array, feeding buzz

## Abstract

Species of open-space bats that are relatively large, such as bats from the genus *Nyctalus*, are considered as high-risk species for collisions with wind turbines (WTs). However, important information on their behaviour and movement ecology, such as the locations and altitudes at which they forage, is still fragmentary, while crucial for their conservation in light of the increasing threat posed by progressing WT construction. We adopted two different methods of microphone array recordings and GPS-tracking capturing data from different spatio-temporal scales in order to gain a complementary understanding of the echolocation and movement ecology of *Nyctalus aviator*, the largest open-space bat in Japan. Based on microphone array recordings, we found that echolocation calls during natural foraging are adapted for fast flight in open-space optimal for aerial-hawking. In addition, we attached a GPS tag that can simultaneously monitor feeding buzz occurrence, and confirmed that foraging occurred at 300 m altitude and that the flight altitude in mountainous areas is consistent with the turbine conflict zone, suggesting that the birdlike noctule is a high-risk species in Japan. Further investigations on this species could provide valuable insights into their foraging and movement ecology, facilitating the development of a risk assessment regarding WTs.

## 1. Introduction

The progressing construction of wind turbines (WTs) is of global concern [1], as especially high-flying bats, e.g. from the genus *Nyctalus* (*Nyctalus lasiopterus* [2] and *Nyctalus noctula* [3]) suffer from fatalities, inflicted near WT blades, and die in great numbers each year [4–8]. The birdlike noctule *Nyctalus aviator*, is distributed in eastern China, Korea and Japan [9], and is listed as vulnerable in the Japanese Red List [10]. With a forearm length of 57–64 mm and an aspect ratio of approximately 6.7, it is one of the largest bat species in Japan [11,12]. Its echolocation call type can range from quasi-constant frequency (QCF) calls to steep frequency-modulated (FM) calls, and are also reported to be of low peak frequency, long pulse length and possibly high sound pressure. Yet, how these bats modulate their echolocation calls during foraging is not known in detail [12,13]. Given these pieces of knowledge, *N. aviator* can be categorized as an open-space, aerial-hawking bat. However, detailed information on its foraging and movement behaviour as well as the altitude of foraging and commuting is not available, yet very important for their conservation in light of the increasing threat posed by progressing WT construction.

Therefore, this study aims at: (i) providing details on movement and foraging behaviour of *N. aviator* and testing its similarity to other *Nyctalus* species, and (ii) demonstrating relationships between the bats' behaviour and spatial features of the WTs. To meet the first aim, we investigate the echolocation call structure, the movement behaviour and habitat use of individual free-ranging bats. Since *N. aviator* bats are similar in size and wing morphology to other *Nyctalus* species [12,14,15], we hypothesize that they will exhibit similar echolocation call structure, movement and foraging behaviour. In particular, we expect to find relatively long quasi-constant echolocation calls that are adapted to flight in open-space with increasing bandwidth and shorter calls when approaching objects or insects as well as a so-called feeding buzz [16,17], which is a characteristic echolocation sequence that indicates a capture attempt of a flying insect. Concerning movement behaviour and habitat use, we expect to find a relatively fast flight at medium to high altitudes to foraging habitats in open areas, e.g. above fields or forests, with a relatively large distance from the roost, which is characteristic for larger open-space bats [2,3,18–21]. Based on the detailed knowledge of the foraging and movement behaviour, we can meet the second aim of estimating the collision risk for *N. aviator*.

The importance of this research topic in general and the importance of this specific study is highlighted by the fact that especially in Asia wind farms are built in an ever increasing pace to achieve net zero CO_2_ emissions [22]. At the same time, little is known about the bat species and their populations that might be affected by the constructed wind farms as relatively little research is conducted in those areas (e.g. [23,24]). In addition, bat species are not part of protected species lists, such as in China [25], which greatly hinders their management and conservation.

## 2. Methods

### 2.1. Microphone array recordings

Microphone array recordings were conducted on a total of 10 days in 2020 and 2021: 4 and 5 August 2020, 26, 27 and 30 June, 2, 28 and 30 July, and 1 and 2 August 2021 (total 1210 min), at Tokiwa Park in Asahikawa city, Hokkaido, Japan (43°46'28.6″ N, 142°21'27.1″ E), in the proximity of *N. aviator* roosts within hollows of *Ulmus davidiana*. The measurement was started around 21.00 in 2020 and around sunset (approx. 19.00) in 2021, and the recordings were made for about 2–3 h for each day until the batteries ran out.

**Figure 1.**
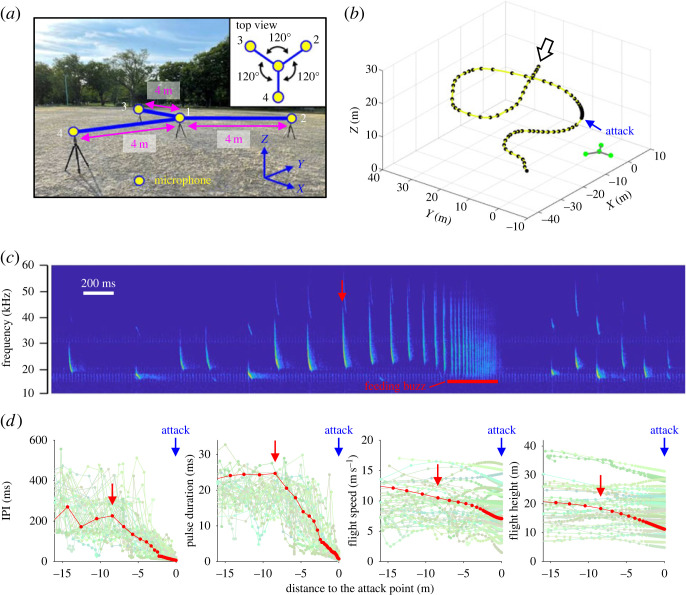
Foraging behaviour of *Nyctalus aviator* recorded by the microphone array. (*a*) Y-shaped four-microphone array arranged 1 m above the ground at Tokiwa Park in Asahikawa city, Hokkaido, Japan. (*b*) Typical foraging path along pulse emission point (black circle) while an attack is identified by feeding buzz sounds. The origin of the axes in the graph is the central microphone of the microphone array. (*c*) Spectrogram of echolocation pulses emitted before and after an attack. The red arrow indicates the starting point of the approach phase (see text). (*d*) Changes in interpulse-interval (IPI), pulse duration, flight speed and flight altitude as a function of distance to the attack point. Data were taken from all echolocation call sequences containing feeding buzzes (*n* = 45 from 38 tracked flights). The red line corresponds to the sound data shown in (*c*). The red arrow indicates the starting point of the approach phase of this data.

A Y-shaped microphone array, consisting of four omnidirectional microphones (FG-23329-C05; Knowles Electronics, Itasca, IL, USA), was placed at a height of approximately 1 m above the ground with the microphone tips pointing upwards (figure 1*a*; electronic supplementary material, appendix S1). Based on the difference in arrival times between a central and three outer microphones, the three-dimensional locations of flying bats were reconstructed using a custom-made program in MATLAB (Math Works, Natick, MA, USA; figure 1*b*). The detailed procedure of recording and calculation of the three-dimensional positions is described in Fujioka *et al.* [26] and Mizuguchi *et al.* [27]. The coordinates of the bats were calculated within a range of 47 m from the microphone array where the theoretical range error was less than 40 cm (corresponding to wing length of *N. aviator*) (electronic supplementary material, appendix S2).

Those echolocation calls that were recorded by the central microphone with a sufficiently high signal to noise ratio were further analysed. The end and start times of the pulses were automatically obtained based on a −15 dB threshold from the peak power. Then, the inter pulse interval (IPI), which is the time between the beginning of a pulse and the end of the next pulse, the pulse duration, the minimum frequency, the bandwidth and the peak frequency were calculated using a custom-made program in MATLAB, respectively. We identified feeding activity based on a characteristic sequence of echolocation calls termed ‘feeding buzz’ from the spectrogram.

### 2.2. Acoustic GPS logging

We caught six bats using mist nets close to a roosting tree (*U. davidiana*) next to Asahikawa elementary school in eastern Asahikawa city, central Hokkaido, Japan (43°46'10.0″ N, 142°26'27.5″ E) during sunset on 29 July 2021. Then, we carefully attached custom-made acoustic GPS data loggers (ArumoTech Corp., Kyoto, Japan, 2.4 g) with a telemetry unit (PicoPip Ag337, Lotek, Canada, 0.3 g) using Skin Bond (Osto-bond, Montreal Ostomy Inc., Canada) to the back of the bat, which is not less than 5%, recommended by Aldridge & Brigham [28]. However, recent GPS studies have confirmed that bats may be able to cope with additional loads exceeding 10% of their body mass without apparent changes to foraging behaviour or body mass [3,19,21,29,30]. We held the bats for about 10 min to allow the glue to dry and released them on the roosting tree.

The timers were set to start GPS logging every 5 s from 20.00 on the second day after the attachment until batteries ran out (approx. 1 h). It is possible to continuously record pulse emission timing with a high temporal resolution of 4 MHz sampling rate, because acoustic GPS loggers are designed to output a high voltage when the acoustic signal voltage exceeds a certain threshold (electronic supplementary material, appendix S3.A).

We identified feeding buzzes from the recorded IPI patterns [31] based on observations from the microphone array recordings. In particular, pulse sequences with at least five pulses below an IPI threshold of 20 ms and a maximum duration of 100 ms were automatically classified as a feeding buzz (attack). The automatic classification of feeding buzzes corresponded to 100% those that we visually found in the IPI sequences. We identified the coordinates of the attack location by resampling GPS data from 5 s to every second using linear completion, and searching for the timing closest to the end of a feeding buzz. The flight altitude was obtained by subtracting the ground altitude (the Geospatial Information Authority of Japan, https://www.gsi.go.jp/ENGLISH/index.html) from the absolute altitude above sea level recorded by the acoustic GPS logger. We identified habitat types (river, mountain, urban) from ‘topographic’ and ‘satellite’ maps in Web Map Service provided by Open Geospatial Consortium via MATLAB (Mapping Toolbox, Mathworks).

### 2.3. Statistical analysis

All statistical analyses were performed in the R environment for statistical computing [32] and its extended packages. We modelled the flight speed, altitude as well as the presence/absence of an attack, as a function of the habitat type (river, mountain, urban area) using linear modelling, generalized linear modelling (lme4, v.1.1-28, [33]) and generalized linear mixed modelling using Template Model Builder (package glmmTMB_1.1.4, [34]), respectively, to examine whether the tagged bat adapted its flight and foraging behaviour to the respective habitat type.

In general, we examined the quality of a model fit graphically using the functions in the DHARMa package (v.0.3.3.0, [35]). We checked whether the model explained more variance than its respective null model by comparing them either via a parametric bootstrapping method (package pbkrtest_0.5.1, [36]) or via a *χ*^2^ test (function ANOVA, R 2022). A *χ*^2^ type-II-Wald test (function ANOVA, package car_3.0.12, [37]) was used to check the significance of the factor within the respective model. Bonferroni correction was applied to all pairwise *post hoc* comparisons between the levels of the factor (function lsmeans, package emmeans_1.8.0) [38].

In the residuals of the linear model for the flight speed, we detected heteroscedasticity which was most probably caused by the strong variance in the data and potentially also by the unbalanced number of data points between habitat categories. To correct the model, we subsampled the data by randomly selecting 76 data points from each habitat category.

The residuals of the first model for flight altitude indicated temporal autocorrelation as well as heteroscedasticity. We corrected this model by randomly subsampling the data to 40 data points per habitat type and applying a glmmTMB model with a Gaussian error distribution and the factor for habitat type as the dispersion parameter.

Finally, we analysed whether the attack probability differed between habitat types by modelling the presence and absence of an attack in a generalized model with binomial error distribution. For this model, we were able to use the full amount of data without any subsampling.

## 3. Results

### 3.1. Echolocation behaviour during foraging

Based on the microphone array recordings, two types of echolocation pulses were mainly observed during the search phase (figure 1*c*); narrow band QCF calls with a relatively long duration and FM calls with a shorter duration (table 1). The approach phase began with a distinct decrease in IPI and pulse duration and an increase in minimum frequency at 8.6 ± 3.2 m (*n* = 45 attacks) before the attack point (figure 1*d*). The feeding buzz emitted just before the attack was characterized by an IPI of 15.0 ± 7.9 ms, lasting for about 251.6 ± 99.2 ms (range: 100.7–523.7 ms).

**Table 1.** Acoustical characteristics of QCF and FM pulses in *N. aviator.* (Pulses of approximately 0.60 kHz ms^−1^ are classified as QCF according to Jones [39] values (3 kHz bandwidth and 5–20 ms pulse duration). Numbers represent mean ± s.d.)

	pulse duration (ms)	bandwidth (kHz)	minimum frequency (kHz)	peak frequency (kHz)
QCF (*n* = 1163)	20.9 ± 3.6	7.78 ± 1.8	15.3 ± 1.5	19.0 ± 1.7
FM (*n* = 499)	14.5 ± 5.0	13.7 ± 3.5	19.0 ± 1.6	23.6 ± 2.5

The flight speed during foraging was 8.21 ± 2.5 m s^−1^ in the search phase and 8.34 ± 3.1 m s^−1^ in the approach phase. The flight height was 16.3 ± 6.0 m during the search phase, 17.6 ± 6.2 m during the approach phase and 17.5 ± 6.3 m during the feeding buzz. The maximum flight height at the end of the feeding buzz was 31 m.

### 3.2. Foraging at high altitudes

**Figure 2.**
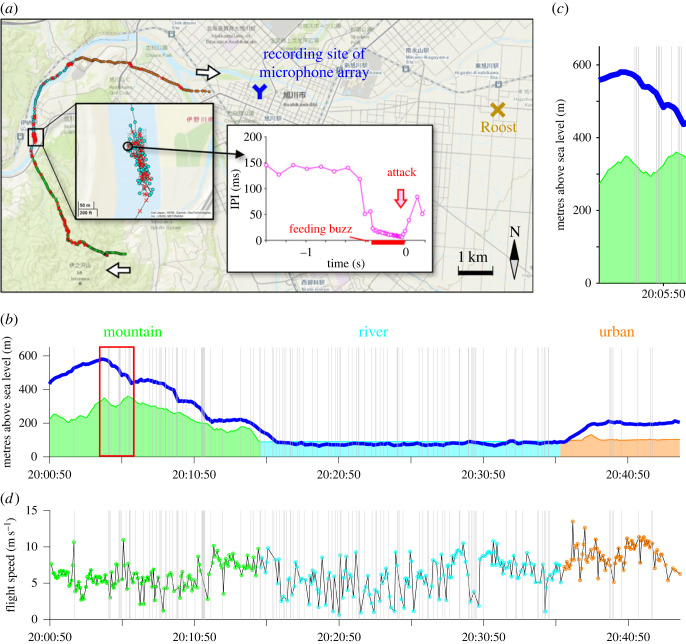
Summary of results based on acoustic GPS logger tracking of an adult female. (*a*) Map shows the 40 min long flight trajectory including attack points (red cross) determined by feeding buzz occurrences. White arrows indicate the flight direction of the bat. The middle inset-graph shows a typical temporal change in IPI before an attack. The location of the exemplary attack point that was selected for this graph is circled in black in the black bordered inset-map. (*b*) Temporal change in flight altitude (blue line) above sea level. Grey vertical lines indicate attack points. (*c*) Enlarged view of (*b*) (indicated by the red square). (*d*) Temporal change in flight speed.

We caught six bats (two juvenile males and four adult females) weighing 44.4 ± 3.5 g (mean ± s.d.). The total weight of the attached logger and the telemetry unit corresponded to 5.4–6.5% of the bat's weight (range: 40.0–49.4 g). We recaptured two bats and successfully collected data from one logger which was attached to an adult female (initial body weight: 44.0 g, weight at recapture: 46.8 g). The acoustic GPS logger recorded from 20. 00 to 20.44 a total of 371 GPS locations over a distance of 16 km from which we identified 120 feeding buzzes (figure 2*a*).

**Figure 3.**
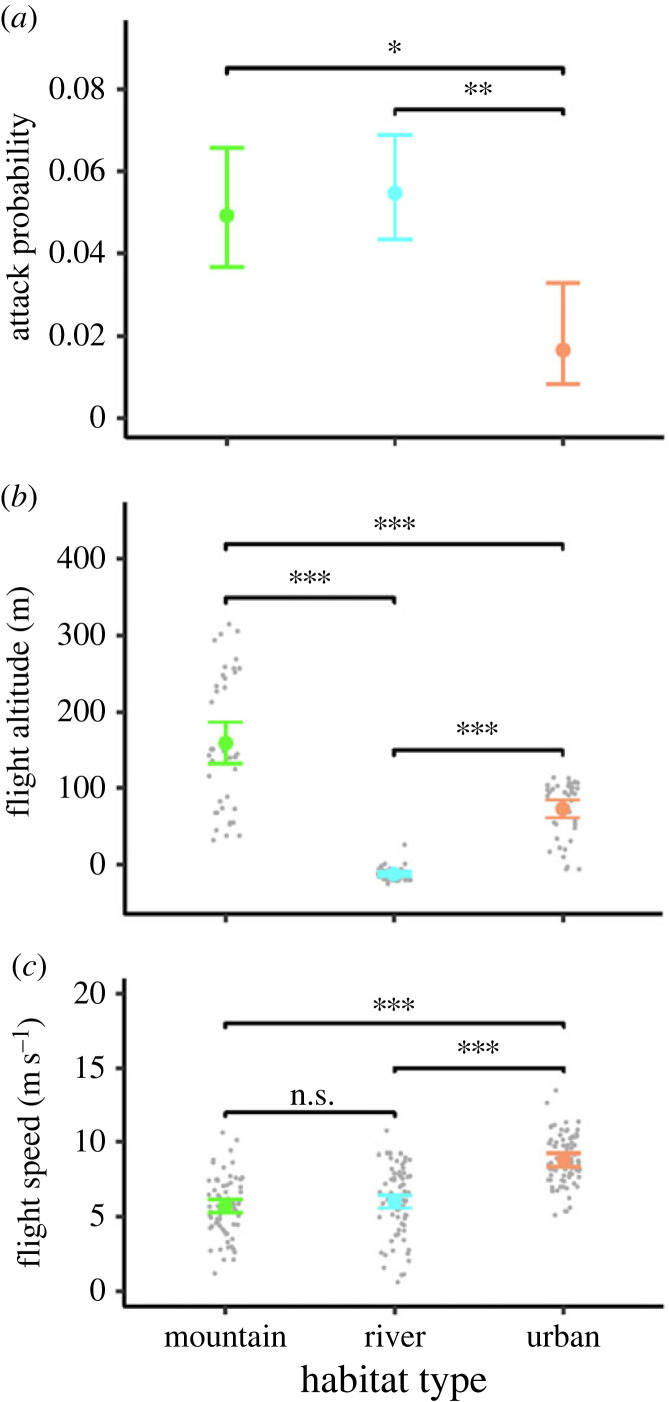
Graph shows the model-based results on (*a*) attack probability, (*b*) flight altitude and (*c*) flight speed as a function of the habitat type. Coloured circles indicate the estimated means and probabilities, respectively, with coloured whiskers indicating the 95% confidence intervals. Grey dots represent the raw data points. Raw data (presence/absence data) were not plotted in (*a*) to ensure optimal representation of model results. Asterisks indicate statistically significant differences (***p < 0.001; **p < 0.01; *p < 0.05)

The bat attacked predominantly over rivers with a total of 69 (58.0%) attacks. With 43 (35.8%) attacks over mountainous areas it attacked less often while it attacked least over urban areas with eight (6.7%) attacks. Similarly, the attack rate (number of attacks per minute) was highest over rivers (3.28 attacks min^−1^), followed by 2.96 attacks min^−1^ over mountains and 1 attack min^−1^ over urban areas. The model of the attack probability as a function of the habitat type (*n* = 2616 overall model significance (PBtest): stat = 14.7, *p* < 0.01; $\chi_{\mathrm{typeII}-\mathrm{Waldtest}}^{2}=14.7$, d.f. = 2, *p* < 0.001), confirmed these patterns and showed that the bat was significantly least probable to attack above the urban area (tables 2 and 3; figure 3) compared to a higher, but not significantly different probability to attack above the mountains. Note that, since the sample size of this study was very low, the statistical model that we constructed only describes patterns within an individual (same for the statistical model below).

**Table 2.** Summary shows estimated means, standard errors, degrees of freedom as well as upper and lower 95% confidence levels for models for flight altitude and speed, whereas probabilities and asymptotic lower and upper confidence levels are given for the model for attack probability. (inf, infinite.)

models for	habitat	estimated means/probability	standard error	degrees of freedom	lower confidence level	upper confidence level
attack probability	mountain	0.049	0.007	inf	0.037	0.066
	river	0.055	0.006	inf	0.043	0.069
	urban	0.017	0.006	inf	0.008	0.033
flight altitude (m)	mountain	159	14	114	132	187
	river	−12	1	114	−15	−10
	urban	73	6	114	62	85
flight speed (m s^−1^)	mountain	5.73	0.22	228	5.29	6.16
	river	6.05	0.22	228	5.61	6.48
	urban	8.85	0.22	228	8.41	9.28

**Table 3.** Overview shows pairwise comparisons among habitat types within specified models that are also indicated in figure 3. (inf, infinite.)

model for	contrast	estimate/odds ratio	standard error	degrees of freedom	*t*-/*z*-ratio	*p-*value
attack probability	mountain/river	0.89	0.18	inf	−0.56	1
	mountain/urban	3.07	1.19	inf	2.88	0.012
	river/urban	3.43	1.29	inf	3.27	0.003
flight altitude (m)	mountain–river	172	14	114	12.33	<0.001
	mountain–urban	86	15	114	5.72	<0.001
	river–urban	−86	6	114	−14.16	<0.001
flight speed (m s^−1^)	mountain–river	−0.32	0.31	228	−1.02	0.9
	mountain–urban	−3.12	0.31	228	−9.96	<0.001
	river–urban	−2.8	0.31	228	−8.94	<0.001

We recorded the highest flight altitudes of 157.7 ± 92.3 m (range: 29.0–317.7 m) above mountains, while the bat flew lower above the urban area (77.1 ± 31.2 m), and the river (−11.1 ± 13.5 m) (figure 2*b*). The model for flight altitude as a function of habitat type (*n* = 120, overall model significance (ANOVA): *χ*^2^_2_ = 136.02, *p* < 0.001; $\chi_{\mathrm{typeII}-\mathrm{Waldtest}}^{2}=344.63$, d.f. = 2, *p* < 0.001), confirmed these patterns and showed that the flight altitude differed significantly between habitat types showing lowest flight altitude at the river with −12.3 ± 1.4 m, higher altitudes above the urban area with 73.2 ± 5.9 m and highest flight altitudes above mountains with 159.3 ± 13.8 m.

The bat attacked at altitudes of 120.7 ± 64.1 m (range: 35.4–306.4 m; figure 2*c*) above mountains while it attacked at altitudes of 99.5 ± 7.7 m and −9.43 ± 14.4 m above urban areas and rivers, respectively. Note that, in general, the altitudinal GPS error is greater than latitudinal and longitudinal errors [21]. In this study, the false negative flight altitudes were obtained at about 153 GPS locations (97.4% of them above the river, −13.8 ± 5.5 m), corresponding to 41.2% of the total locations. This indicates that our study also expects a negative error of at least 0.7–26.1 m in the flight altitude measurement.

When flying over the urban area, the flight speed recorded by the GPS logger was 8.8 ± 1.7 m s^−1^ and faster than 6.0 ± 1.8 m s^−1^ over the mountains and 6.1 ± 2.4 m s^−1^ over the river (figure 2*d*). The respective linear model (*n* = 228 overall model significance (PBtest): stat = 97.7, d.f. = 2, *p* < 0.001; *F* = 69.5, d.f. = 2, *p* < 0.001), confirmed that the flight speed above the urban area with 8.9 ± 0.2 m s^−1^ was significantly faster compared to both, above the river with 6.1 ± 0.2 m s^−1^ and the mountains with 5.7 ± 0.2 m s^−1^.

## 4. Discussion

The highest altitude that we recorded for an attack was 306.4 m above mountains, which, to our knowledge, indicates for the first time that *N. aviator* was foraging while flying at high altitudes of 300 m. Collision with WTs is one of the main causes of multiple mortality events in bats around the world [1]. The flight altitude of *N. aviator* in mountainous areas is consistent with the turbine conflict zones (e.g. 30–130 m above the ground), which is a concern in other studies focused on species from the genus *Nyctalus* [18,19,21,40,41]. Actually, there are some cases that the birdlike noctule collided with the WTs in Japan [42]. Our study demonstrated that the birdlike noctule flew at the height of the WT, suggesting that they are a high-risk species in Japan. However, sample size in this study was very low (*n* = 1). Further investigations on this bat species could provide valuable insights into their foraging and movement ecology, facilitating the development of a risk assessment with regard to WT.

High-altitude flight has been reported by GPS tagging in several bat species, including *Nyctalus* species; flight from 124 m to 367 m above ground in *N. lasiopterus* [2] and scouting flights to 100 m and a maximum of 300 m above ground in *N. noctula* [18]. The reasons for high-altitude flight by *Nyctalus* species have not been well elucidated, but it is suggested that insectivorous bats may be attracted to high altitudes, i.e. they may prey on migratory insects that match weather conditions to increase flight cost efficiency for both insects and bats [43]. High-altitude flight could also facilitate advanced movements that could use landmarks for visual navigation and aid in effective foraging and navigation [2,40,44]. Probably, in *Nyctalus* species, they may opportunistically prey on migratory birds with high energy balance while flying at high altitudes [45].

On the other hand, the frequent low-altitude feeding buzzes over the river may be because of the possibility of drinking water as well as foraging. The tagged bat was a lactating female which requires a large amount of water, especially during the lactation period. The fact that the highest concentration of foraging was observed in the river area suggests the possibility that *N. aviator* feed on aquatic insects that are concentrated near the water's surface [46], or that the flat surface of the water without any obstructions facilitates echolocation. In the future, it would be necessary to increase sample size and then investigate behavioural characteristics according to sex and season by comparing with males and non-lactating females.

The flight speed of *N. aviator* measured by GPS was the fastest during flight in urban areas, probably because of the commuting to the roost. On the other hand, the average flight speed while foraging, as measured by microphone array, was faster than that of the other Vespertilionidae, i.e. 5 m s^−1^ of the Japanese house bat *Pipistrellus abramus* [47]. *Nyctalus aviator* bats are also characterized by long and narrow wings and low frequency, high intensity echolocation calls [12,13]. In addition to the low frequency and high intensity of echolocation calls that are conventionally considered to be the characteristics of echolocation in *Nyctalus* species [15], we found that the long IPI and pulse duration, as well as the long approach starting distance to attack the prey, are other characteristics of echolocation suitable not only for high-speed flight, but also suitable for detection of relatively large and fast airborne targets at great distances.

In this study, we proposed a method for combining measurement data at different scales, namely microphone array and acoustic GPS logger, and reported new findings on high-altitude foraging by bats through this method, which will contribute to a complementary understanding of the echolocation and migration ecology of the bat species.

## Data Availability

The datasets generated and analysed during the current study are available from the Dryad Digital Repository: https://doi.org/10.5061/dryad.mgqnk993n [48]. The data are provided in the electronic supplementary material [49].
